# Impact of barometric pressure on adhesive small bowel obstruction: a retrospective study

**DOI:** 10.1186/s12893-020-00829-1

**Published:** 2020-07-25

**Authors:** Yuta Yamamoto, Yusuke Miyagawa, Masato Kitazawa, Hirokazu Tanaka, Masatsugu Kuroiwa, Nao Hondo, Makoto Koyama, Satoshi Nakamura, Shigeo Tokumaru, Futoshi Muranaka, Yuji Soejima

**Affiliations:** Division of Gastroenterological, Hepato-Biliary-Pancreatic, Transplantation and Pediatric Surgery, Department of Surgery, Shinshu University School of Medicine, 3-1-1 Asahi, Matsumoto, Nagano, 390-8621 Japan

**Keywords:** Adhesive small bowel obstruction, Barometric pressure, Fasting, Decompression, Surgery, Reciprocal fluctuation

## Abstract

**Background:**

Adhesive small bowel obstruction (ASBO) is one of the most common causes of postoperative morbidity. According to Boyle’s law, decreased barometric pressure expands the volume of intestinal gas. We aimed to elucidate the relationship between barometric pressure and ASBO.

**Methods:**

We divided 215 admissions of 120 patients with ASBO into three groups: the fasting group, which responded to fasting (*n* = 51); the decompression group, which was successfully treated with gastrointestinal decompression (*n* = 104); and the surgery group which required emergency or elective surgery to treat ASBO (*n* = 60). We compared and examined clinical backgrounds, findings on admission, and barometric pressure during the peri-onset period (29 days: from 14 days before to 14 days after the onset of ASBO).

**Results:**

There were significant differences among the three groups regarding gender, history of ASBO, hospital length of stay, and barometric pressure on the onset day of ASBO. Barometric pressure on the onset day was significantly higher in the fasting group than in the decompression group (*p* = 0.005). During pre-onset day 5 to post-onset day 2, fluctuations in the barometric pressure in the fasting and decompression groups showed reciprocal changes with a symmetrical axis overlapping the median barometric pressure in Matsumoto City; the fluctuations tapered over time after onset. In the fasting group, the barometric pressure on the onset day was significantly higher than that on pre-onset days 14, 11, 7, 4, 3, and 2; post-onset days 3 and 10; and the median pressure in Matsumoto City. Conversely, in the decompression group, the barometric pressure on the onset day was lower than that on pre-onset days 14, 5–2; post-onset days 1, 2, 7, 8, 11, 13, and 14; and the median pressure in Matsumoto City. In the surgery group, the barometric pressure on the onset day was equivalent to those on the other days.

**Conclusions:**

ASBO with response to conservative treatment is vulnerable to barometric pressure. Additionally, ASBO that is successfully treated with fasting and decompression is associated with a different barometric pressure on the onset day and reciprocal fluctuations in the barometric pressure during the peri-onset period.

## Background

Adhesive small bowel obstruction (ASBO) is one of the most common causes of postoperative morbidity. It occurs in 3% of all laparotomies; 1% of patients undergo surgery for ASBO within 1 year after undergoing laparotomy [1–3]. Patients with previous abdominal surgery sometimes develop ASBO despite intentionally consuming easily digestible food and chewing well. Although climate change has been previously reported to be associated with the onset of ASBO [4], it has remained unclear as to whether any other factors other than diet could induce ASBO. The management of ASBO is based on clinical parameters including history, physical examination, laboratory analysis, and computed tomography (CT) imaging. Recent advances in the diagnostic imaging technology of contrast CT have enabled us to accurately identify the findings that indicate intestinal ischemia, including decreased bowel wall enhancement, mesenteric edema, and the closed-loop sign [5–7]. Some patients with ASBO are successfully treated with only fasting, whereas others require intestinal decompression through nasogastric tube (NGT) and long-tube (LT) placement. When conservative management fails, or when patients show signs indicating intestinal ischemia, surgical intervention is necessary. Nevertheless, discrimination of diverse ASBO in respect to the response to each treatment remains unclear.

Several diseases have been reported to be related to barometric pressure, including acute ischemic stroke [8], benign paroxysmal positional vertigo [9], and migraine headache [10]. According to Boyle’s law, it is assumed that decreased barometric pressure causes the volume of intestinal gas to expand. Nevertheless, the relationship between ASBO and barometric pressure has not yet been clarified. Therefore, this study aimed to determine the relationship between barometric pressure and ASBO.

## Methods

### Patients and study design

**Fig. 1:**
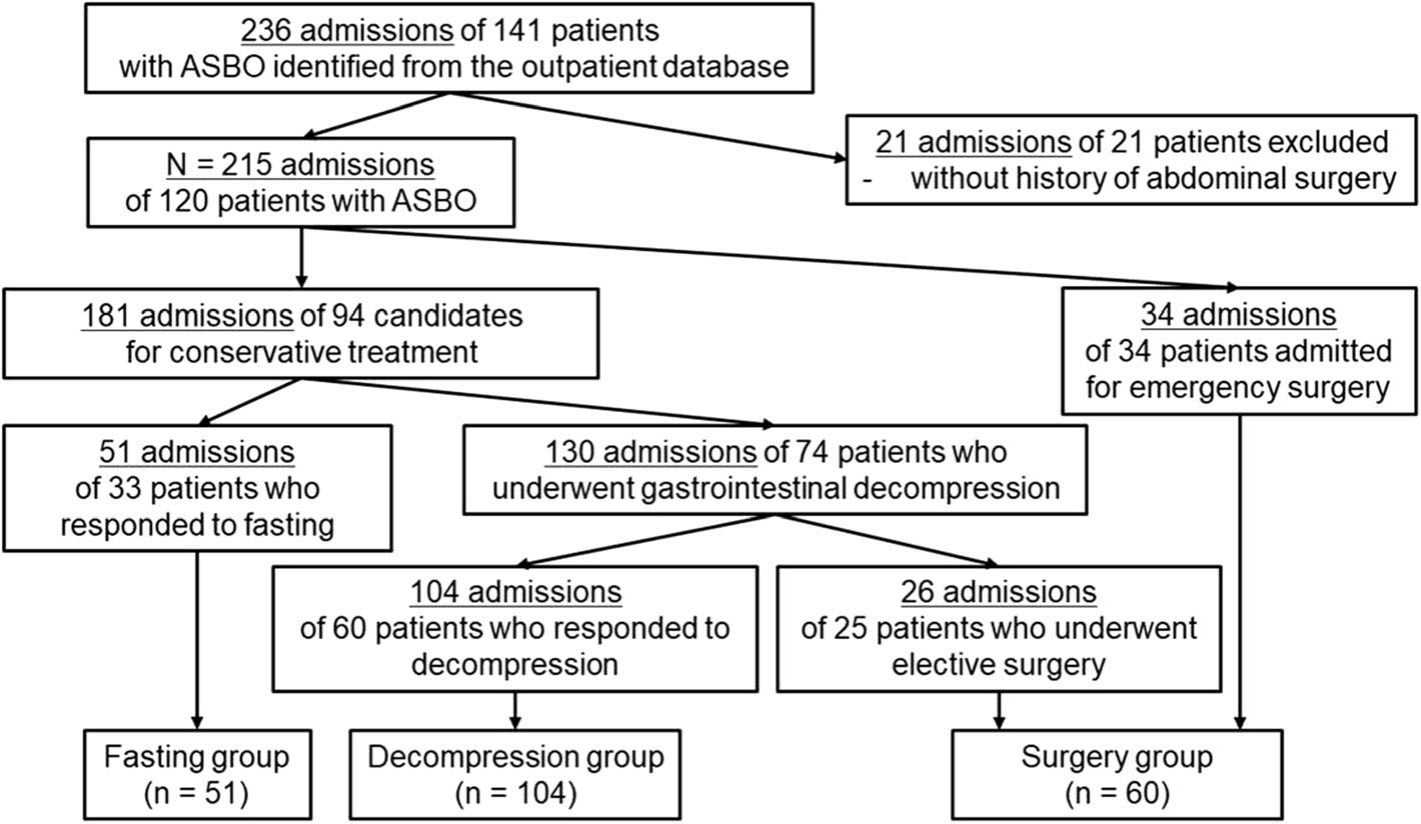
Flowchart of the selection of study groups. ASBO, adhesive small bowel obstruction

This retrospective cohort study included patients with ASBO who were admitted to Shinshu University Hospital between November 2007 and August 2019. ASBO was diagnosed by clinical symptoms including abdominal pain, nausea, and vomiting, as well as radiological imaging that demonstrated a dilated small intestine with a diameter > 2.5 cm. During the inclusion period, 141 patients (236 admissions) with ASBO were admitted to our department. We excluded 21 admissions of 21 patients who had no history of abdominal surgery. Our final study group consisted of 120 patients (215 admissions) who were divided into three groups: the fasting group, which responded to fasting (*n* = 51); the decompression group, which was successfully treated with gastrointestinal decompression (*n* = 104); and the surgery group, which required emergency or elective surgery to treat ASBO (*n* = 60) (Fig. 1). We compared and examined clinical backgrounds, findings on admission, and barometric pressure during the peri-onset period (29 days: from 14 days before to 14 day after the onset of ASBO). With regard to the management of ASBO, intravenous fluids were administered to all patients. Initially, we assessed the requirement of emergency surgery including strangulation and ischemia or congestion of the small intestine. When these findings were not confirmed, the patients were judged to be candidates for conservative treatment. In general, patients with improving clinical symptoms on admission were managed with fasting in the first 24–48 h. When the obstruction did not improve, they were treated with gastrointestinal decompression including NGT, hyperbaric oxygen therapy (HBO), and LT. Other candidates for conservative treatment with active symptoms were treated with gastrointestinal decompression at first. In cases in which the obstruction continued for more than 1 week, or when the obstruction returned after diet resumption, we performed elective surgery.

Shinshu University Hospital is located in Matsumoto City, Nagano prefecture, almost in the center of Honshu, a main island in Japan, at an altitude of 610 m. Data regarding the average daily barometric pressure in Matsumoto City were obtained from the website of the Japan Meteorological Agency (URL: www.jma.go.jp/jma/index.html). The data were the average of the pressure readings, which were automatically, continuously measured from 0:00 to 24:00 each day using the Automated Meteorological Data Acquisition System at the Matsumoto Meteorological Station.

### Statistical analysis

Statistical analysis was performed using the Statistical Package for the Social Sciences version 23.0 (SPSS; Chicago, IL, USA). Demographic data are presented with descriptive statistics. Non-parametric data are presented as medians with interquartile ranges. With regard to the barometric pressure in each group during the peri-onset period, parametric and non-parametric data were intermingled; thus, we considered all barometric pressure to be non-parametric data. Comparisons between qualitative variables were performed using the Chi-square test. The Kruskal-Wallis test was used to compare non-parametric data among the three groups. If a significant difference (*p* < 0.05) was found, the Mann-Whitney U test with a Bonferroni correction for multiple comparisons was used. The Wilcoxon matched-pairs signed-rank test was used to compare the median barometric pressure on the onset day to that on another day in each group. The one-sample Wilcoxon signed rank test was used to determine whether a median barometric pressure on a certain day in each group differed from the median barometric pressure in Matsumoto City from November 2007 to August 2019 (943.4 hPa). All tests were two-tailed, and differences with a *p*-value of < 0.05 were considered statistically significant.

## Results

The characteristics of patients in each group are shown in Additional File 1. There were significant differences among the three groups in terms of gender (*p* = 0.007), history of ASBO (*p* < 0.001), hospital length of stay (LOS) (*p* < 0.001), barometric pressure on pre-onset day 1 (*p* = 0.049), and on ASBO onset day (*p* = 0.006). As a result of the Mann-Whitney U test with a Bonferroni correction, LOS was shorter in the fasting group than in the decompression (*p* = 0.002) and surgery groups (*p* < 0.001), and LOS was shorter in the decompression group than in the surgery group (*p* < 0.001). Barometric pressure on the onset day was significantly higher in the fasting group than in the decompression group (*p* = 0.005) (Table 1).

**Table 1:** *P-*values of the Mann-Whitney U test with a Bonferroni correction for multiple comparisons

	Fasting vs Decompression	Fasting vs Surgery	Decompression vs Surgery
Hospital length of stay	0.002*	< 0.001*	< 0.001*
Barometric pressure			
Pre-onset day 1	0.052	1.000	0.475
Onset day	0.005*	0.553	0.239

Asterisks indicate statistical significance (*p* < 0.05)

**Fig. 2:**
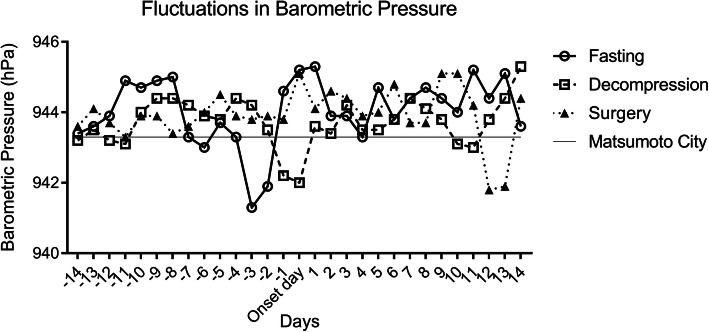
Fluctuations in the median barometric pressure in each subgroup during the peri-onset period. A horizontal line on 943.4 hPa indicates the median barometric pressure in Matsumoto City from November 2007 to August 2019

With regard to fluctuation in barometric pressure in each subgroup during the peri-onset period, the line graph shows a reciprocal change in the fasting and decompression groups, in particular from pre-onset day 5 to post-onset day 2, with a symmetrical axis overlapping the median barometric pressure in Matsumoto City from November 2007 to August 2019 (Fig. 2). In line with this, the barometric pressure on the onset day was significantly higher than those on pre-onset days 4–2 in the fasting group, whereas the pressure on the onset day was lower than those on pre-onset days 5–2 in the decompression group (Table 2). Additionally, these two lines tapered over time after the onset. Compared to the median barometric pressure in Matsumoto City, the pressures on the onset day and post-onset day 1 were significantly higher in the fasting group (*p* = 0.011 and 0.008, respectively), whereas that on the onset day was significantly lower in the decompression group (*p* = 0.049) (Table 3). These findings showed that ASBO, which responds to fasting and decompression, is associated with reciprocal fluctuations in barometric pressure like the sine curve during the peri-onset period, in particular from pre-onset day 5 to post-onset day 2, with the amplitude representing significant change in the barometric pressure on the onset day. However, in the surgery group, the barometric pressure on the onset day was equivalent to those on the other days (Table 2) and the median barometric pressure in Matsumoto City (Table 3).

**Table 2:** Comparison of the barometric pressures between onset and another day in each group

	Fasting group			Decompression group			Surgery group		
	Barometric pressure (hPa)	Difference (hPa)	*p-*value	Barometric pressure (hPa)	Difference (hPa)	*p-*value	Barometric pressure (hPa)	Difference (hPa)	*p-*value
Pre-onset day 14	943.6	−1.6	0.029*	943.2	1.2	0.031*	943.6	−1.5	0.363
Pre-onset day 13	943.6	−1.6	0.109	943.5	1.5	0.214	944.1	−1.0	0.988
Pre-onset day 12	943.9	−1.3	0.097	943.2	1.2	0.160	943.7	−1.4	0.877
Pre-onset day 11	944.9	−0.3	0.047*	943.1	1.1	0.240	943.3	−1.8	0.491
Pre-onset day 10	944.7	−0.5	0.211	944.0	2.0	0.080	943.9	−1.2	0.697
Pre-onset day 9	944.9	−0.4	0.609	944.4	2.3	0.064	943.9	−1.2	0.836
Pre-onset day 8	945.0	−0.2	0.228	944.4	2.4	0.068	943.4	−1.7	0.752
Pre-onset day 7	943.3	−1.9	0.041*	944.2	2.2	0.103	943.6	−1.5	0.977
Pre-onset day 6	943.0	−2.3	0.087	943.9	1.9	0.159	944.0	−1.1	0.563
Pre-onset day 5	943.7	−1.5	0.157	943.8	1.8	0.027*	944.5	−0.6	0.683
Pre-onset day 4	943.3	−1.9	0.038*	944.4	2.4	0.003*	943.9	−1.2	0.546
Pre-onset day 3	941.3	−3.9	< 0.001*	944.2	2.2	0.009*	943.8	−1.3	0.780
Pre-onset day 2	941.9	−3.4	0.013*	943.5	1.5	0.006*	943.9	−1.2	0.768
Pre-onset day 1	944.6	−0.6	0.283	942.2	0.2	0.322	943.8	−1.3	0.877
Onset day	945.2(Control)	–	–	942.0(Control)	–	–	945.1(Control)	–	–
Post-onset day 1	945.3	0.1	0.970	943.6	1.5	0.024*	944.1	− 1.0	0.924
Post-onset day 2	943.9	−1.3	0.232	943.4	1.3	0.026*	944.6	−0.5	0.282
Post-onset day 3	943.9	−1.3	0.023*	944.2	2.1	0.062	944.4	−0.7	0.688
Post-onset day 4	943.3	−2.0	0.063	943.5	1.5	0.167	943.9	−1.2	0.724
Post-onset day 5	944.7	−0.5	0.066	943.5	1.5	0.287	944.0	−1.1	0.831
Post-onset day 6	943.8	−1.5	0.060	943.8	1.8	0.192	944.8	−0.3	0.473
Post-onset day 7	944.4	−0.9	0.065	944.4	2.3	0.019*	943.6	−1.5	0.686
Post-onset day 8	944.7	−0.6	0.130	944.1	2.0	0.029*	943.7	−1.4	0.836
Post-onset day 9	944.4	−0.8	0.087	943.8	1.8	0.171	945.1	0.0	0.184
Post-onset day 10	944.0	−1.3	0.042*	943.1	1.0	0.067	945.1	0.0	0.132
Post-onset day 11	945.6	0.4	0.357	943.0	1.0	0.013*	944.2	−0.9	0.949
Post-onset day 12	944.6	−0.6	0.398	943.8	1.8	0.051	941.8	−3.3	0.137
Post-onset day 13	945.1	−0.1	0.302	944.4	2.4	0.003*	941.9	−3.2	0.156
Post-onset day 14	943.6	−1.6	0.176	945.3	3.3	< 0.001*	944.4	−0.7	0.592

Asterisks indicate statistical significance (*p* < 0.05)

**Table 3:** Barometric pressure comparison between the study groups and the median barometric pressure in Matsumoto City

	Fasting group		Decompression group		Surgery group	
	Barometric pressure (hPa)	*p-*value	Barometric pressure (hPa)	*p-*value	Barometric pressure (hPa)	*p-*value
Pre-onset day 14, median (IQR)	943.6 (939.0–947.0)	0.564	943.2 (940.0–948.2)	0.447	943.6 (938.9–946.4)	0.448
Pre-onset day 13, median (IQR)	943.6 (939.2–948.6)	0.970	943.5 (939.1–947.4)	0.876	944.1 (940.6–946.0)	0.651
Pre-onset day 12, median (IQR)	943.9 (940.1–947.5)	0.626	943.2 (940.1–946.6)	0.928	943.7 (941.2–947.2)	0.383
Pre-onset day 11, median (IQR)	944.9 (938.9–947.3)	0.899	943.1 (940.0–946.4)	0.662	943.3 (940.1–947.2)	0.836
Pre-onset day 10, median (IQR)	944.8 (940.7–948.7)	0.158	944.0 (940.3–947.4)	0.588	943.9 (939.5–948.4)	0.766
Pre-onset day 9, median (IQR)	945.1 (940.7–948.5)	0.076	944.4 (939.4–947.4)	0.517	943.9 (939.9–947.3)	0.642
Pre-onset day 8, median (IQR)	945.0 (941.0–947.1)	0.212	944.4 (939.4–947.8)	0.513	943.4 (940.6–947.8)	0.303
Pre-onset day 7, median (IQR)	943.3 (938.7–946.7)	0.981	944.2 (940.1–947.6)	0.433	943.6 (940.2–947.1)	0.648
Pre-onset day 6, median (IQR)	943.1 (938.7–948.3)	0.910	943.9 (939.2–947.8)	0.879	944.0 (940.6–947.2)	0.245
Pre-onset day 5, median (IQR)	943.7 (939.4–947.1)	0.761	943.8 (939.5–948.7)	0.521	944.5 (939.7–946.9)	0.664
Pre-onset day 4, median (IQR)	943.4 (938.7–947.4)	0.968	944.4 (940.0–947.7)	0.181	943.9 (940.4–948.4)	0.433
Pre-onset day 3, median (IQR)	941.3 (936.8–946.6)	0.073	944.2 (940.2–948.5)	0.179	943.8 (938.7–948.1)	0.889
Pre-onset day 2, median (IQR)	942.0 (939.3–947.6)	0.746	943.5 (940.2–947.7)	0.357	943.9 (938.0–948.2)	0.892
Pre-onset day 1, median (IQR)	944.6 (940.9–949.6)	0.111	942.2 (938.9–946.2)	0.095	943.8 (939.9–947.4)	0.656
Onset day, median (IQR)	945.2 (941.9–949.9)	0.010*	942.0 (937.3–946.8)	0.047*	945.1 (938.5–947.6)	0.642
Post-onset day 1, median (IQR)	945.3 (940.3–950.4)	0.024*	943.6 (938.4–946.7)	0.498	944.1 (939.7–947.6)	0.538
Post-onset day 2, median (IQR)	943.9 (941.0–949.0)	0.131	943.4 (939.6–948.1)	0.827	944.6 (940.7–948.3)	0.166
Post-onset day 3, median (IQR)	944.0 (938.1–947.5)	0.609	944.2 (939.0–947.4)	0.851	944.4 (940.7–947.2)	0.317
Post-onset day 4, median (IQR)	943.3 (938.4–947.7)	0.786	943.5 (939.6–947.6)	0.981	943.9 (939.7–946.9)	0.958
Post-onset day 5, median (IQR)	945.0 (938.7–947.6)	0.929	943.5 (939.3–947.4)	0.811	944.0 (938.7–947.7)	0.839
Post-onset day 6, median (IQR)	944.0 (941.0–946.4)	0.677	943.8 (938.7–946.7)	0.626	944.8 (939.4–948.8)	0.284
Post-onset day 7, median (IQR)	944.4 (941.4–947.7)	0.592	944.4 (940.2–947.2)	0.185	943.7 (939.9–949.3)	0.522
Post-onset day 8, median (IQR)	944.9 (939.6–949.0)	0.494	944.1 (940.3–947.6)	0.290	943.7 (939.3–947.9)	0.632
Post-onset day 9, median (IQR)	944.6 (938.3–947.6)	0.948	943.8 (939.8–947.0)	0.957	945.1 (940.6–948.6)	0.095
Post-onset day 10, median (IQR)	944.0 (937.2–948.9)	0.859	943.1 (940.0–946.7)	0.896	945.1 (939.3–948.5)	0.154
Post-onset day 11, median (IQR)	945.6 (938.9–949.2)	0.197	943.0 (940.5–947.0)	0.897	944.2 (937.8–948.8)	0.721
Post-onset day 12, median (IQR)	944.6 (940.7–949.6)	0.095	943.8 (939.2–947.9)	0.953	941.8 (937.9–948.8)	0.096
Post-onset day 13, median (IQR)	945.1 (940.2–948.2)	0.298	944.4 (939.8–948.3)	0.225	941.9 (938.6–945.5)	0.101
Post-onset day 14, median (IQR)	943.6 (940.1–947.8)	0.466	945.3 (940.1–949.6)	0.013*	944.4 (940.9–947.6)	0.096

Asterisks indicate statistical significance (*p* < 0.05). IQR, interquartile range. The median pressure in Matsumoto City was 943.4 hPa

## Discussion

We found that barometric pressure was associated with ASBO which responded to conservative treatment during the peri-onset period. In detail, barometric pressure on the onset day was significantly higher in the fasting group than in the decompression group, and barometric pressures in both groups were significantly different from the median pressure in Matsumoto City. Additionally, the fluctuations in barometric pressure during the peri-onset period in the two groups were significant and reciprocal. When considering the unknown etiology and difficulty of predicting the response and failure of conservative management, these results have significant importance because they suggest the possible impact of barometric pressure on the etiology and treatment of ASBO. Based on our results, observation of the fluctuations in pressure may predict the incidence of ASBO and response to conservative treatment because of the unique fluctuation during the peri-onset period. Additionally, there is a possibility that patients with ASBO who do not need gastrointestinal decompression treatment can be identified. This is important as it can prevent them from the risk of respiratory complications, as nasogastric decompression significantly increases pneumonia and respiratory failure in patients with ASBO [11].

In Japan, HBO is regarded as an optional treatment for many diseases which require emergency treatment, including carbon monoxide poisoning, sudden sensorineural hearing loss, central retinal vein occlusion, and ASBO, although few clinical trials have addressed its role in ASBO [12–14]. Based on Boyle’s law, decreased barometric pressure causes the intestinal gas volume to expand, and conversely, increases in pressure cause intestinal decompression. Because it remains unclear whether the effect of HBO on ASBO in humans is cellular, biochemical, or physical in nature, it is difficult to explain the mechanism of our result. However, some animal experiments have been performed [15–17]. In an experiment on dogs that used intestinal closed loop obstructions, Cross reported that, as barometric pressure increased, the absorption of gas from the closed loop obstructions increased in dogs who were breathing in ambient air (i.e., not breathing in a high concentration of oxygen) [18]. In that experiment, an average of 10.4% of the injected air diffused from the loops after 24 h at 1 atm pressure, and 27.0% was absorbed after the same number of hours at 2 atm pressures, with an increase of 16.6%. Even though natural variation in barometric pressure may have a small impact on the intestinal absorption of gas, the influence of the pressure on ASBO does not simply depend on the physical properties of intestinal gas.

There are several limitations to this study. First, it was a single-center study and therefore may be subject to selection bias. Second, the number of patients was too small to adequately determine the relationship between barometric pressure and ASBO; thus, this may result in a beta error. Third, the parameter that we used did not perfectly represent the exact barometric pressure at specific times and places in patients with ASBO. In particular, not all patients lived in Matsumoto City, and the barometric pressure that we used in this study was the 24 h mean value; further, we did not consider circadian change. Finally, although we found that barometric pressure affected ASBO in some way, it is still unclear whether the pressure affects the etiology or treatment, or both.

Therefore, prospective multi-center studies incorporating larger patient populations are needed to draw definite conclusions on whether barometric pressure is associated with the etiology and treatment of ASBO.

## Conclusions

Barometric pressure on the onset day was significantly higher in the fasting group than in the decompression group. Additionally, the fluctuations in barometric pressure during the peri-onset period in the two groups were significant and reciprocal.

## Supplementary information

**Additional file 1.** Patients’ demographics and summary of data. Patients’ demographics and summary of data by study group.

## Data Availability

The datasets used and/or analyzed during the current study are available from the corresponding author on reasonable request.

## Abbreviations

ASBO: adhesive small bowel obstruction
CT: computed tomography
NGT: nasogastric tube placement
LT: long-tube placement
HBO: hyperbaric oxygen therapy
LOS: length of stay
