# Supplementary material for: Impact of barometric pressure on adhesive small bowel obstruction: a retrospective study
Source: BMC Surg. 2020 Jul 25;20:168. doi: 10.1186/s12893-020-00829-1 (PMC7382815; doi:10.1186/s12893-020-00829-1)
Supplement: Supplementary file 1 — Additional file 1. Patients’ demographics and summary of data. Patients’ demographics and summary of data by study group. [file 12893_2020_829_MOESM1_ESM.docx]

| Additional file 1 Patients' demographics and summary of data | | | | | |
| --- | --- | --- | --- | --- | --- |
|  |  | Fasting  (n = 51) | Decompression  (n = 104) | Surgery  (n = 60) | *p-*value |
| Gender | |  |  |  |  |
|  | Male (%) | 32 (62.7) | 82 (78.8) | 34 (56.7) | 0.007* |
|  | Female (%) | 19 (37.3) | 22 (21.2) | 26 (43.3) |  |
| Age (years) | |  |  |  |  |
|  | Median (IQR) | 69.0 (59.0-76.0) | 71.0 (63.0-79.0) | 75.0 (65.8-83.0) | 0.059 |
| Body mass index | |  |  |  |  |
|  | Median (IQR) | 19.4 (18.3-22.3) | 20.1 (18.1-22.9) | 19.8 (17.6-21.7) | 0.294 |
| Performance status | |  |  |  |  |
|  | 0, 1 (%) | 43 (84.3) | 98 (94.2) | 52 (86.7) | 0.104 |
|  | 2, 3, 4 (%) | 8 (15.7) | 6 (5.8) | 8 (13.3) |  |
| Primary disease | |  |  |  |  |
|  | Benign (%) | 14 (27.5) | 21 (20.2) | 20 (33.3) | 0.167 |
|  | Esophagus/Stomach malignancy (%) | 10 (19.6) | 33 (31.7) | 11 (18.3) | 0.095 |
|  | Colorectal malignancy (%) | 15 (29.4) | 33 (31.7) | 14 (23.3) | 0.517 |
|  | Other malignancy (%) | 12 (23.5) | 17 (16.3) | 15 (25.0) | 0.344 |
| Surgical approach for primary disease | |  |  |  |  |
|  | Laparotomy (%) | 47 (92.2) | 100 (96.2) | 58 (96.7) | 0.459 |
|  | Laparoscopy (%) | 4 (7.8) | 4 (3.89) | 2 (3.3) |  |
| History of ASBO | |  |  |  |  |
|  | Yes (%) | 34 (66.7) | 67 (64.4) | 19 (31.7) | <0.001* |
|  | No (%) | 17 (33.3) | 37 (35.6) | 41 (68.3) |  |
| History of chemotherapy | |  |  |  |  |
|  | Yes (%) | 14 (33.3) | 27 (26.0) | 12 (20.0) | 0.603 |
|  | No (%) | 37 (67.3) | 77 (74.0) | 48 (80.0) |  |
| History of radiotherapy | |  |  |  |  |
|  | Yes (%) | 46 (90.2) | 100 (96.2) | 58 (96.7) | 0.218 |
|  | No (%) | 5 (9.8) | 4 (3.89) | 2 (3.3) |  |
| White blood cell count (/µl) | |  |  |  |  |
|  | Median (IQR) | 7600 (6040-10200) | 8760 (6948-11995) | 8790 (6833-11860) | 0.152 |
| Length of stay (day) | |  |  |  |  |
|  | Median (IQR) | 9 (6-13) | 13 (9-17) | 24 (13-34) | <0.001* |
| Barometric pressure (hPa) | |  |  |  |  |
|  | Pre-onset day 14, median (IQR) | 943.6 (939.0-947.0) | 943.2 (940.0-948.2) | 943.6 (938.9-946.4) | 0.484 |
|  | Pre-onset day 13, median (IQR) | 943.6 (939.2-948.6) | 943.5 (939.1-947.4) | 944.1 (940.6-946.0) | 0.944 |
|  | Pre-onset day 12, median (IQR) | 943.9 (940.1-947.5) | 943.2 (940.1-946.6) | 943.7 (941.2-947.2) | 0.694 |
|  | Pre-onset day 11, median (IQR) | 944.9 (938.9-947.3) | 943.1 (940.0-946.4) | 943.3 (940.1-947.2) | 0.846 |
|  | Pre-onset day 10, median (IQR) | 944.8 (940.7-948.7) | 944.0 (940.3-947.4) | 943.9 (939.5-948.4) | 0.663 |
|  | Pre-onset day 9, median (IQR) | 945.1 (940.7-948.5) | 944.4 (939.4-947.4) | 943.9 (939.9-947.3) | 0.523 |
|  | Pre-onset day 8, median (IQR) | 945.0 (941.0-947.1) | 944.4 (939.4-947.8) | 943.4 (940.6-947.8) | 0.909 |
|  | Pre-onset day 7, median (IQR) | 943.3 (938.7-946.7) | 944.2 (940.1-947.6) | 943.6 (940.2-947.1) | 0.887 |
|  | Pre-onset day 6, median (IQR) | 943.1 (938.7-948.3) | 943.9 (939.2-947.8) | 944.0 (940.6-947.2) | 0.631 |
|  | Pre-onset day 5, median (IQR) | 943.7 (939.4-947.1) | 943.8 (939.5-948.7) | 944.5 (939.7-946.9) | 0.994 |
|  | Pre-onset day 4, median (IQR) | 943.4 (938.7-947.4) | 944.4 (940.0-947.7) | 943.9 (940.4-948.4) | 0.698 |
|  | Pre-onset day 3, median (IQR) | 941.3 (936.8-946.6) | 944.2 (940.2-948.5) | 943.8 (938.7-948.1) | 0.075 |
|  | Pre-onset day 2, median (IQR) | 942.0 (939.3-947.6) | 943.5 (940.2-947.7) | 943.9 (938.0-948.2) | 0.738 |
|  | Pre-onset day 1, median (IQR) | 944.6 (940.9-949.6) | 942.2 (938.9-946.2) | 943.8 (939.9-947.4) | 0.049* |
|  | Onset day, median (IQR) | 945.2 (941.9-949.9) | 942.0 (937.3-946.8) | 945.1 (938.5-947.6) | 0.006* |
|  | Post-onset day 1, median (IQR) | 945.3 (940.3-950.4) | 943.6 (938.4-946.7) | 944.1 (939.7-947.6) | 0.066 |
|  | Post-onset day 2, median (IQR) | 943.9 (941.0-949.0) | 943.4 (939.6-948.1) | 944.6 (940.7-948.3) | 0.503 |
|  | Post-onset day 3, median (IQR) | 944.0 (938.1-947.5) | 944.2 (939.0-947.4) | 944.4 (940.7-947.2) | 0.804 |
|  | Post-onset day 4, median (IQR) | 943.3 (938.4-947.7) | 943.5 (939.6-947.6) | 943.9 (939.7-946.9) | 0.981 |
|  | Post-onset day 5, median (IQR) | 945.0 (938.7-947.6) | 943.5 (939.3-947.4) | 944.0 (938.7-947.7) | 0.923 |
|  | Post-onset day 6, median (IQR) | 944.0 (941.0-946.4) | 943.8 (938.7-946.7) | 944.8 (939.4-948.8) | 0.468 |
|  | Post-onset day 7, median (IQR) | 944.4 (941.4-947.7) | 944.4 (940.2-947.2) | 943.7 (939.9-949.3) | 0.956 |
|  | Post-onset day 8, median (IQR) | 944.9 (939.6-949.0) | 944.1 (940.3-947.6) | 943.7 (939.3-947.9) | 0.966 |
|  | Post-onset day 9, median (IQR) | 944.6 (938.3-947.6) | 943.8 (939.8-947.0) | 945.1 (940.6-948.6) | 0.356 |
|  | Post-onset day 10, median (IQR) | 944.0 (937.2-948.9) | 943.1 (940.0-946.7) | 945.1 (939.3-948.5) | 0.437 |
|  | Post-onset day 11, median (IQR) | 945.6 (938.9-949.2) | 943.0 (940.5-947.0) | 944.2 (937.8-948.8) | 0.661 |
|  | Post-onset day 12, median (IQR) | 944.6 (940.7-949.6) | 943.8 (939.2-947.9) | 941.8 (937.9-948.8) | 0.059 |
|  | Post-onset day 13, median (IQR) | 945.1 (940.2-948.2) | 944.4 (939.8-948.3) | 941.9 (938.6-945.5) | 0.085 |
|  | Post-onset day 14, median (IQR) | 943.6 (940.1-947.8) | 945.3 (940.1-949.6) | 944.4 (940.9-947.6) | 0.541 |
| Asterisks indicate statistical significance. IQR, interquartile range; ASBO, adhesive small bowel obstruction. | | | | | |
